# Sperm DNA fragmentation after radioiodine treatment for differentiated thyroid cancer

**DOI:** 10.1186/s12610-015-0024-1

**Published:** 2015-08-02

**Authors:** Camille Esquerré-Lamare, François Isus, Nathalie Moinard, Louis Bujan

**Affiliations:** Université de Toulouse, UPS, Groupe de Recherche en Fertilité Humaine (EA 3694, Human Fertility Research Group), TSA 70034, 31059, Toulouse, Cedex 9 France; CECOS Midi-Pyrénées, Groupe d’Activité de Médecine de la Reproduction, Paule de Viguier University Hospital, Toulouse, France

**Keywords:** Radioiodine therapy, SCSA, Sperm DNA fragmentation, Differentiated thyroid, Cancer, Semen characteristics

## Abstract

**Background:**

Treatment of differentiated thyroid cancer usually consists of a total thyroidectomy followed by one or several courses of radioiodine (^131^I). ^131^I is known to have deleterious effects on radiation sensitive tissues and irradiation to the testes has been shown after its administration. We investigated effects of such treatment on sperm DNA in a patient with differentiated thyroid carcinoma.

**Methods:**

The patient, a 32-year-old male with differentiated thyroid carcinoma treated by total thyroidectomy and radioiodine therapy, performed 6 semen samples in total, 3 for sperm banking and 3 for semen exploration, that were analysed for classic semen parameters. DNA integrity was analysed by flow cytometry: sperm DNA fragmentation index (DFI) and high DNA stainability (HDS) were analyzed by sperm chromatin structure assay, DNA fragmentation was analyzed by terminal deoxynucleotidyl transferase dUTP nick end labeling assay.

**Results:**

Moderate oligozoospermia was observed as early as 3 months after a first dose of ^131^I and became severe at 5 months. Total sperm count was reduced up to 12 months after the second dose of ^131^I. Sperm DFI was increased 3.25 months after the first dose of ^131^I. All parameters returned to normal values 28 months after the second ^131^I dose.

**Conclusions:**

Treatment with ^131^I induces alterations in sperm chromatin as well as in sperm parameters a short time (3 months) after a first dose of ^131^I with persistence of sperm alterations until 12 months after a second dose. Sperm banking should be recommended before treatment.

## Résumé

**Contexte:**

Le traitement du cancer différencié de la thyroïde consiste en général en une thyroïdectomie totale suivie d’un traitement par l’iode radioactif (^131^I). L’^131^I est connu pour ses effets délétères sur les tissus sensibles aux radiations et il a également été démontré une irradiation des testicules après administration d’iode radioactif. Nous avons donc cherché à savoir quels étaient les effets du traitement par l’iode radioactif sur l’ADN des spermatozoïdes.

**Méthodes:**

Le patient est un homme de 32 ans présentant un cancer différencié de la thyroïde traité par thyroïdectomie totale puis traitement par iodothérapie. Il a réalisé 6 prélèvements de sperme, 3 pour de l’autoconservation et 3 pour le suivi après traitement, dont les caractéristiques spermatiques ont été étudiées suivant les méthodes classiques. L’analyse de l’intégrité de la chromatine a été réalisée par cytométrie en flux : l’index de fragmentation de l’ADN (DFI) et la haute colorabilité de l’ADN (HDS) ont été analysés par le sperm chromatin structure assay (SCSA), la fragmentation de l’ADN a été évaluée par la technique de terminal deoxynucleotidyl transferase dUTP nick end labeling (TUNEL).

**Résultats:**

Une oligozoospermie modérée a été observée dès 3 mois après une première dose de ^131^I, et est devenue sévère à 5 mois. La numération des spermatozoïdes est restée réduite jusqu’à 12 mois après la seconde dose de ^131^I. Le DFI est élevé 3.25 mois après la première dose de ^131^I. Tous les paramètres ont retrouvé des valeurs normales 28 mois après la seconde dose d’^131^I.

**Conclusion:**

Le traitement par ^131^I induit des altérations de la chromatine du spermatozoïde en plus de celles des caractéristiques spermatiques une courte période (3mois) après une première dose de ^131^I, avec persistance de ces altérations spermatiques jusqu’à 12 mois après une seconde dose. L’autoconservation devrait être recommandée avant un tel traitement.

**Mots clé:**

Traitement par iode radioactif, SCSA, fragmentation de l’ADN du spermatozoïde, cancer différencié de la thyroïde, paramètres spermatiques.

## Background

Differentiated thyroid cancer (DTC) is the most common endocrine malignancy. It affects any age group and sex, and its incidence has been increasing for the last 20 years [1, 2]. Treatment of DTC includes thyroidectomy, followed by nuclear medicine therapy with radioiodine ^131^I for eradication of persistent disease and ablation of residual thyroid remnant, to facilitate post-treatment radiological and clinical follow-up of the patient [3]. ^131^I treatment can damage radiation-sensitive tissues, and it has been shown that some radiation is delivered to the testes after administration of radioiodine [4]. Some studies have reported impairment in spermatogenic functions such as elevated serum FSH levels, reduced sperm count resulting in some cases in oligozoospermia [5–8]. These effects were usually not permanent and were reversed within 18 months, although permanent damage has been reported in patients receiving repeated or high cumulative doses [9]. Radiations are known to induce double strand breaks in DNA, and it has been shown that treatment with ^131^I induces a rise in γH2AX and 53BP1 DNA repair foci in blood cells [10]. There are no reports on potential effects on spermatozoa DNA, although it has been shown that higher levels of DNA strand breaks are associated with infertility and miscarriages [11].

We report the case of a 32-year-old man in whom several sperm analyses were carried out after treatment with ^131^I for differentiated thyroid carcinoma. Each sample was analyzed for classic semen parameters. For the first time, we report the effects of radioiodine therapy on spermatozoa chromatin and DNA, evaluated by sperm chromatin structure assay (SCSA) and terminal deoxynucleotidyl transferase dUTP nick end labeling (TUNEL) assay.

### Subject

**Fig. 1:**
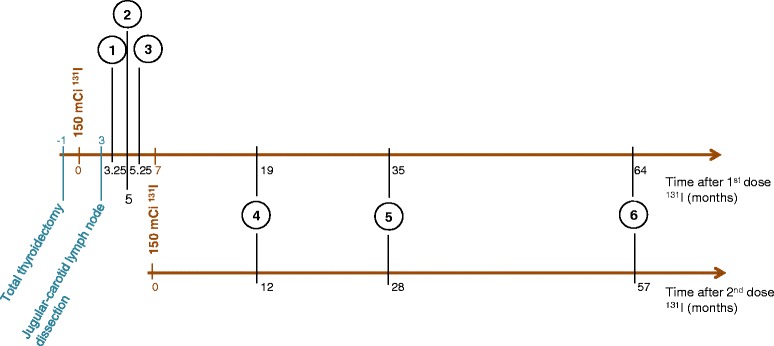
Semen exploration according to time after surgery and ^131^I treatments The upper line represents time in months after the first ^131^I treatment, the lower line time in months after the second ^131^I treatment. Numbers in circles represent ejaculates, ex: 1 = 1st ejaculate. **150 mCi** ^**131**^ **I** represents the two treatments by ^131^I. Total thyroidectomy and Jugular-carotid lymph node dissection represent the two surgeries the patient underwent

A 32-year-old man presented with thyroid carcinoma classified pT3N1R1. One month after a total thyroidectomy, he was treated with 150 mCi of radioiodine (^131^I). A second dose of 150 mCi ^131^I was administered 7 months later. The patient was referred to the CECOS for sperm banking 3 months after the first dose of ^131^I and before the scheduled second dose. He was seen twice during the two months after his first sperm banking, and three times after his second dose of ^131^I, at 12, 28 and 57 months. The patient gave a total of 6 samples, 3 for sperm banking and 3 for exploration (Fig. 1). The present study was carried out in accordance with the Declaration of Helsinski and the patient gave his informed consent for the study. All samples and exposure data were provided by the GERMETHEQUE Biobank (France).

### Semen analysis

Sperm samples were collected by masturbation after 3–6 days of sexual abstinence, except for sample 1 where abstinence was 30 days. Semen was allowed to liquefy 30 min at 37 °C before parameters were analyzed. Conventional semen analysis was performed according to WHO criteria [12]. Sperm count (SC), total sperm count (TSC), motility a + b (rapidly progressive—grade a, slowly progressive—grade b) (MOT) were assessed. Sperm vitality (VIT) was determined by eosin-nigrosin. Semen samples were cryopreserved within 1 h of collection according to the standard procedures used for sperm banking in our laboratory until later processing for testing DNA integrity [13].

### Spermatozoa fixation

Prior to SCSA and TUNEL, cells were fixed with 1 % paraformaldehyde. Briefly, spermatozoa were collected by thawing straws of the different samples at room temperature. Cryoprotector medium was washed by adding 5 ml of PBS medium, drop by drop to 1 ml then more rapidly for the remaining 4 ml, and centrifuging at 630 g for 10 min. Cells we resuspended in 1 ml of PBS and 4 ml of 1 % paraformaldehyde and incubated 30 min at room temperature. Samples were then centrifuged 10 min at 1500 g, the supernatant discarded and cells resuspended in the appropriate volume of PBS. Samples were kept at 4 °C until processed. A minimum of 13 × 10^6^ spermatozoa were needed for SCSA and TUNEL analyses. In case of oligozoospermia (ie 5 months after Iodotherapy) only SCSA test was possible on 4 × 10^6^ spermatozoa.

### Sperm chromatin structure assay

Sperm DFI and high DNA stainability (HDS) were measured according to conventional sperm chromatin structure assay (SCSA) as described by Evenson and Jost [14] and routinely used in our laboratory. In brief, 2 × 10^6^ cells were centrifuged at 1500 g for 10 min and transferred into a cytometer tube. Each sample was run in duplicate. Cells were resuspended by vortexing, then 0.4 ml of acid detergent (pH 1.2) was added. The tube was gently shaken for 30 s before adding 1.2 ml of acridine orange and incubated 3 min on ice. 5,000 cells were acquired by fluorescence-activated cell sorting (FACS) on a FC500 cytometer (Beckman Coulter Inc., Fullerton, CA, USA). The DNA fragmentation index (DFI) was defined as the ratio of denatured DNA (red fluorescence) to total DNA (red + green fluorescence). High DNA stainability (HDS) was calculated as the percentage of cells presenting a high level of red fluorescence.

### DNA strand break measurement

DNA strand breaks were measured using the terminal deoxynucleotidyl transferase dUTP nick end labeling (TUNEL) assay. A detailed protocol for the TUNEL assay of human sperm has previously been described [15]. Terminal deoxynucleotidyl transferase (TdT) enzyme, reaction buffer, CoCl2 and dUTP biotin were purchased from Roche Diagnostics (Mannheim, Germany). Streptavidin FITC was purchased from Tebu-Bio (Le Perray-en-Yvelines, France), and propidium iodide from Sigma Aldrich (St Louis, MO, USA). For each specimen, a negative sample without enzyme and two positive samples with enzyme were tested. The percentage of cells presenting DNA strand breaks was calculated by subtracting the percentage of fluorescence in the control (no TdT enzyme) from the mean percentage of fluorescence of the two TdT-positive samples. 10,000 cells were acquired by FACS on a FC500 cytometer (Beckman Coulter Inc., Fullerton, CA, USA).

## Results

### Treatment by ^131^I induces perturbations in semen parameters

The patient was seen 6 times for sperm banking and evaluation of semen parameters. The first 3 samples were obtained 3.25, 5 and 5.25 months after the first dose of ^131^I. The other samples were obtained 12, 28 and 57 months after the second dose (Fig. 1). Results of analysis of semen parameters are shown in Table 1. Three months and 1 week after the first dose of radioiodine, moderate oligozoospermia (SC and TSC) was observed, and became severe at 5 and 5.25 months after the first treatment. The percentage of progressive motile spermatozoa was lower than normal values after the first dose of ^131^I, 3.25 (20 %) and 5.25 months post-treatment (30 %).

**Table 1:** Sperm characteristics and DNA fragmentation values evaluated by TUNEL assay after ^131^I treatments

	Time after 1st dose (months)			Time after 2nd dose (months)		
	3.25	5	5.25	12	28	57
Sexual abstinence (days)	30	4	4	6	3	5
Volume (ml)	1.3	1.6	1.45	1.6	1.55	2.1
Sperm count (× 10^6^/ml)	18	4	3.5	20	100	183
Total sperm count (× 10^6^)	23.4	6.4	5.1	32	155	384.3
Motility a + b (%)	20	35	30	35	50	40
Viability (%)	51	45	67	62	83	65
TUNEL value (%)	10.5	ND	ND	3.4	5.1	3.35

Normal values (WHO laboratory manual for the examination and processing of human semen, 5th edition, 2010): volume > 1.5 ml, sperm count > 15 × 10^6^/ml, total sperm count > 39 × 10^6^/ejaculate, motility > 32 %, viability > 58 %. ND: not determined

TSC was lower than normal values 12 months after the second dose of radioiodine (32 × 10^6^ spermatozoa). At twenty-eight months, SC, TSC, MOT and VIT returned to normal values. By 57 months post-treatment, all semen parameters were normal.

### DNA fragmentation

**Fig. 2:**
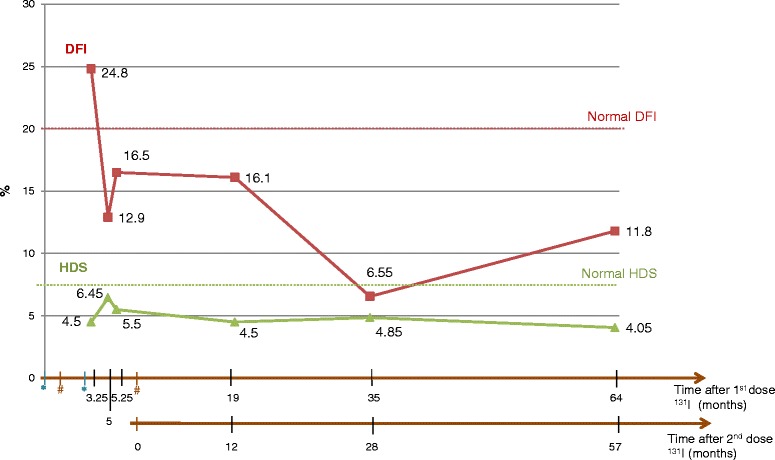
Variation of sperm DNA fragmentation after ^131^I treatment DNA Fragmentation Index (DFI) () and High DNA Stainability (HDS) () were measured on 2 x 10^6^ cells by the SCSA assay. Each dot represents the mean of duplicates for each sample, values are reported on the graph.  represents surgeries,  represents ^131^I treatments. The Y axis represents the values in % of DFI and HDS

The DNA fragmentation index (DFI) measured by SCSA was under the threshold value of 20 %, defined in our laboratory [16] as the 90th percentiles of SCSA values of 51 fertile men, for all samples except for specimen 1 collected 3.25 months after the first dose of ^131^I, where DFI was highest (24.5 %) (Fig. 2).

Values decreased to 12.9 % and 16.5 % at 5 and 5.25 months after the first dose. Twelve months after the second dose of ^131^I the value remained stable at 16.1 %. The lowest values were found at 28 and 57 months after the second dose of radioiodine (6.55 % and 11.8 %, respectively). HDS values remained constant during the whole study period (around 4 %), under the threshold value of 7.5 %, with a slight elevation to 6.45 % at 5.25 months after the first dose (Fig. 2).

The percentage of cells with fragmented DNA assessed by TUNEL was in the normal range during the study, i.e. <16.5 % [16] (Table 1), although the highest value was found 3.25 months after the first dose of ^131^I (10.5 %). The technique could not be performed in samples at 5 and 5.25 months after the first dose of radioiodine due to an insufficient number of spermatozoa.

## Discussion

A few studies have reported the effects of radioiodine therapy for thyroid cancer on sperm characteristics [5–7, 17–19] but none has analyzed the consequences of such treatment on sperm DNA. For the first time, we report a transient sperm DNA fragmentation increase 3.25 months after administration of a single dose of 150 mCi of radioiodine. Our case report shows major alterations of sperm characteristics 3.25, 5 and 5.25 months after a single dose of 150 mCi radioiodine, consisting of moderate to severe oligozoospermia, moderate to severe asthenospermia, necrospermia and hypospermia. It is noteworthy that very severe effects on sperm production were also noted 3–6 months after the end of treatment of testicular cancer by adjuvant radiotherapy, although the irradiation mode was totally different [16]. These observed alterations of sperm characteristics are consistent with the findings of several studies that show a decrease in total sperm count and normokinetic sperm in patients treated with 30 mCi to more than 600 mCi of ^131^I, as summarized in Table 2. Moreover, an increase in FSH as early as 3 months after doses of 150–700 mCi of ^131^I has been reported by Wichers et al. [6], with a maximum value 6 months after therapy and recovery of the pre-treatment mean value by 18 months. To date, no study has reported results concerning potential damage to spermatozoa chromatin and DNA although it is known that treatment with ^131^I induces radiation to the testes [4], mainly through contamination of blood and bladder urine. Radiation is known to induce alterations in cell DNA, so it makes sense to assume that radiation issued from treatment by ^131^I can affect spermatozoa DNA. Our results show that DFI and DNA fragmentation values were highest 3.25 months after the first dose of ^131^I, and then decreased at a distance from the second dose. These results show abnormal chromatin and fragmented DNA, testifying that radioiodine affects not only classic sperm parameters but also the genetic material of spermatozoa. We assume these consequences may be due to the radiation, although we cannot rule out a possible effect of the tumor context itself. Unfortunately, we have no sperm sample obtained before radiation, so we cannot be certain that this patient’s sperm DNA parameters were normal. But we do know that this patient fathered two children without difficulty (Time to pregnancy, 3 months), and his excellent sperm parameters at a distance from the end of therapy (28 and 57 months) lead us to think that all parameters were normal prior to his treatment. One of the limits of our study is that we were not able to investigate the possible epigenetic effects of ^131^I radiation. Potential modifications might be expected, as it has recently been shown that rats exposed to 20 Gy of X-ray to the brain, and whose body was protected by a shield, present a decrease in DNA methylation in testicular tissue as well as in mature sperm cells [20]. Another study on health workers occupationally exposed to ionizing radiations shows an increase in DNA methylation in the spermatozoa of these men [21]. Unfortunately we lack the material to answer such a question but it would be of interest to investigate this aspect in future studies.

**Table 2:** Published effects of one or several doses of ^131^I on sperm parameters and serum FSH levels

Authors	Number of patients	Dose of ^131^I (mCi)	Time after ^131^I (months)	FSH serum concentration (IU/l)	Sperm parameters
			0	32	azoospermia
Handelsman et al., 1980 [17]	1	350	5	16	
			9	18	
Handelsman and Turtle, 1983 [5]	5 7	223 ± 53 1 dose ≤ 100 multiple doses ≥ 100	median 12 (1–42)	no elevation ↑	6 men performed sperm analysis: 2 azoospermic (doses 400 and 350 mCi)4: 11 × 10^6^ ≤ SC ≤ 32 × 10^6^
Pacini et al., 1994 [19]	40	30–100	6–12	12.3 ± 7.5	9 men performed sperm analysis: 8 have minor to evident reduced sperm motility
	24	101–200		14.2 ± 9.6	
	22	201–400		15.4 ± 7.4	
	8	401–600		18.9 ± 10.6	
	9	>600		27.7 ± 15.1	
	4	several doses		↑ after each dose-remained elevated after 60 months	
Wichers et al., 2000 [6]	11	150–700	3	17	no data
	18		6	21.3 ± 2.4	
	22		12	13	
	18		18	7.4 ± 1.3	
Hyer et al., 2002 [18]	7	81	1	11–24	no data
			3	8–22	
			6	3–17	
			9	7–10	
	7	2nd dose (dose not specified)		↑ FSH after 2nd dose back to normal by 12 months	
Rosario et al., 2006 [7]	52	100–150	6	24.2 ± 3.2	oligozoospermia in 66% patients with elevated FSH 18 months after treatment
			12	9.5 ± 1.9	
			18	5.9 ± 1.9	

## Conclusions

For the first time, our study shows alterations in sperm chromatin and DNA as well as in sperm parameters a short time (3 months) after a first dose of ^131^I. Sperm characteristics were still altered 12 months after a second dose. Although these results need to be confirmed in more subjects, they should encourage us to do a prospective study to explore genetic and epigenetic modifications on a group of patients who are to receive ^131^I treatment. While awaiting the results of such a study, recommendation for sperm banking before starting radioiodine treatment should be the rule for young patients of reproductive age.

## Abbreviations

DTC: Differentiated thyroid cancer
SCSA: Sperm chromatin structure assay
DFI: DNA fragmentation index
HDS: High DNA stainability
TUNEL: Terminal deoxynucleotidyl transferase dUTP nick end labeling
SC: Sperm count
TSC: Total sperm count
VIT: Sperm vitality
MOT: Sperm motility
